# Oral Verruciform Xanthoma within Lichen Planus: A Case Report and Literature Review

**DOI:** 10.1155/2018/1615086

**Published:** 2018-04-22

**Authors:** Vasileios I. Theofilou, Alexandra Sklavounou, Prokopios P. Argyris, Evanthia Chrysomali

**Affiliations:** ^1^Department of Oral Medicine and Pathology, School of Dentistry, National and Kapodistrian University of Athens, Athens, Greece; ^2^Department of Diagnostic and Biological Sciences, School of Dentistry, University of Minnesota, Minneapolis, MN, USA

## Abstract

**Background:**

Verruciform xanthoma is an uncommon benign tumor, which exhibits a wide range of clinical patterns. The occurrence of the lesion in patients with immune-mediated mucocutaneous diseases may suggest a role of localized epithelial cell damage and chronic inflammation in its pathogenesis.

**Case Report:**

A case of verruciform xanthoma on the tongue of a 56-year-old female with oral lichen planus is reported. An asymptomatic pink-white lesion with a granular surface was observed in the left lateral lingual border, which was closely associated with a white plaque and striae. An incisional biopsy was performed, and histologically, epithelial projections in a verrucous pattern were observed. In the subepithelial connective tissue, aggregates of foamy cells that exhibited immunoreactivity for CD68 were noted. The final diagnosis was verruciform xanthoma. The mucosa adjacent to the lesion demonstrated histopathological features consistent with lichen planus.

**Conclusions:**

A total of twelve cases of oral verruciform xanthomas in patients with oral lichen planus including the present case have been reported in the literature. The clinician should be aware that verruciform xanthoma may mimic malignancy, and therefore, biopsy is required for definitive diagnosis to be established, especially when this tumor develops within conditions that show potential for malignant transformation.

## 1. Introduction

Verruciform xanthoma (VX) is an uncommon benign tumor, which primarily affects the oral mucosa, with the anogenital area and the skin being the second sites affected. It occurs most commonly in white males between 40 and 70 years of age [1–3]. VX exhibits a wide spectrum of clinical presentation including a solitary, well-demarcated, asymptomatic lesion with variable color ranging from pink and red to white, yellowish, or brownish. The clinical features also encompass a papillary or granular roughened surface with a sessile or pedunculated base and delineated slightly raised margins. The center of the lesion may rarely appear crateriform and even ulcerated [3–5]. Microscopically, the hallmark of VX is the presence of foamy (xanthomatous) cells in the connective tissue papillae [1–5].

The pathogenesis of VX remains largely unknown, but the lesion appears to occur in the presence of a localized immune response or inflammation after a local injury [2–4, 6, 7]. Several VX cases have been reported in association with underlying immune-mediated or other conditions, such as pemphigus vulgaris [8], lupus erythematosus [9], dystrophic epidermolysis bullosa [10], graft-versus-host disease (GVHD) [11], epithelial dysplasia and squamous cell carcinoma [1, 12, 13], or congenital hemidysplasia with ichthyosiform erythroderma and limb defects (CHILD) syndrome [14]. The coexistence of VX with lichen sclerosus, lichen planus, or other conditions in the genitalia is well described in the dermatology literature [15], but only 11 cases have been reported in the oral mucosa of patients with the aforementioned disease [4, 7, 13, 16–21].

On the other hand, oral lichen planus (OLP) is a common inflammatory condition affecting 0.1–2.2% of the population, which has been associated with approximately 1% of OLP patients developing oral squamous cell carcinoma based on a recent meta-analysis [22]. However, the potential of OLP malignant transformation is still controversial and yet to be completely clarified.

The clinical diagnosis of VX may be challenging, and the differential diagnosis should include benign lesions, such as squamous papilloma, condyloma acuminatum, and verruca vulgaris, potentially malignant disorders including leukoplakia and its subtypes and erythroplakia, and malignant epithelial tumors, such as verrucous carcinoma and invasive squamous cell carcinoma [1–4]. We report a case of VX occurring in proximity to OLP lesions that showed clinical features suspicious of malignancy.

## 2. Case Presentation

**Figure 1:**
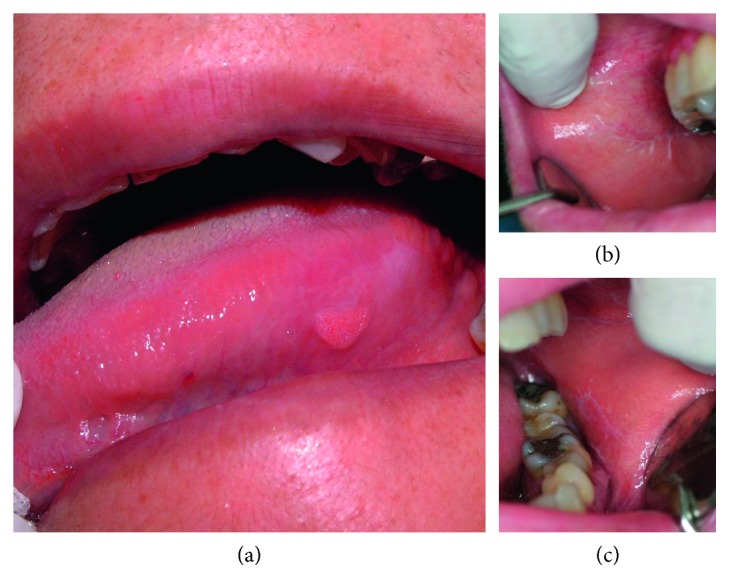
Lingual lesion with slightly raised margins and a granular surface closely associated with a white plaque and striae (a). Whitish striae located on the right (b) and left (c) buccal oral mucosa of the patient.

A 56-year-old female was referred for a painless tongue lesion of three-month duration. The patient had unremarkable medical history, was normolipemic, nonalcohol drinker, smoker (6–19 cigarettes/day) for 30 years, and was taking no medications. On clinical examination, an asymptomatic pink-white, well-demarcated, sessile lesion with a granular surface and slightly raised margins measuring 1 × 0.5 × 0.3 cm was observed in the left lateral lingual border which extended to the ventral surface of the tongue. The lesion was soft in consistency on palpation and closely related to an area of combined white plaque and striae (Figure 1). Similar white striae in a reticular pattern were also observed in the right and left buccal mucosa consistent with the clinical diagnosis of OLP (Figures 1 and 1). There was no evidence of cervical lymph node enlargement. The extraoral examination performed by a dermatologist did not reveal any skin or genital lesions. Regarding the tongue lesion, the possibility of malignancy arising within OLP of the reticular/hypertrophic type was taken under consideration. An incisional biopsy was performed under local anesthesia from a region that included both the granular and the whitish tongue lesions.

**Figure 2:**
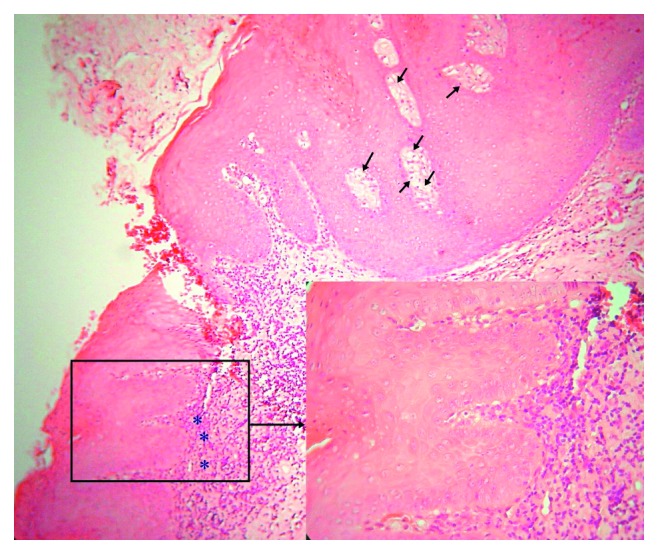
Photomicrograph of the VX that demonstrates hyperparakeratosis with keratin plugs, elongated, thickened epithelial rete ridges, and subepithelial connective tissue filled by foamy cells (arrows). The oral mucosa adjacent to the lesion shows a band-like (asterisks) dense inflammatory infiltrate (hematoxylin and eosin stain, ×150). A higher magnification (inset, ×400) showing epithelial parakeratosis, acanthosis, basal cell hydropic degeneration, and subepithelial predominant lymphocytic infiltrates.

**Figure 3:**
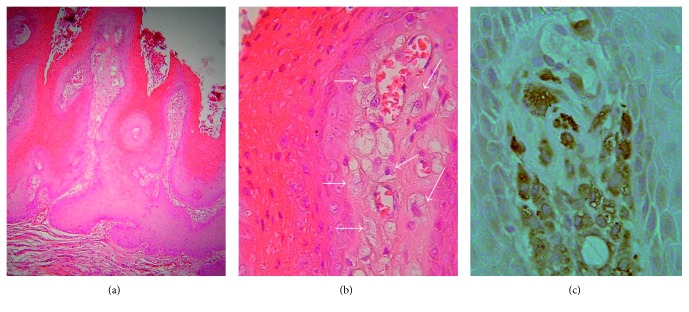
Low-power histologic aspect of the lesion (a) demonstrating exophytic growth in a verrucous/warty pattern with epithelial projections, hyperparakeratosis, orange keratin plugs, and elongated, thickened epithelial rete ridges (hematoxylin and eosin stain ×150). Higher magnification (b) shows aggregates of foamy cells (arrows) in the connective tissue between the rete ridges (hematoxylin and eosin stain ×400), which express (c) strong immunoreactivity for CD68 (immunohistochemical stain, ×400).

Microscopic examination showed hyperparakeratosis and acanthosis with projections of the surface epithelium in a verrucous pattern, intense orange parakeratin plugs, and elongated thickened rete ridges (Figures 2 and 3(a)). Epithelial cell atypia was not evident. Accumulation of foamy cells in the subepithelial connective tissue confined in the lamina propria papillae was noted with sparse inflammatory infiltrates (Figures 2 and 3(b)). The oral mucosa adjacent to the lesion demonstrated histopathological features consistent with lichen planus. Specifically, the epithelial hyperplastic pattern in a transitional manner changed into a relatively thinner squamous epithelium that exhibited parakeratosis, basal cell hydropic degeneration, and a band-like subepithelial dense chronic inflammatory infiltrate mainly by lymphocytes (Figure 2, inset). Based on the clinical and histopathological findings, a final diagnosis of VX with concomitant oral lichen planus features was rendered using the accepted diagnostic criteria for OLP [23].

Immunohistochemical evaluation on formalin-fixed paraffin-embedded tissue sections was performed using CD68 antibody (Dako, Glostrup, Denmark) on a Ventana NexES automated immunohistochemistry system (Ventana Medical Systems, Tucson, AZ). The foamy cells exhibited strong immunostaining for CD68 (Figure 3(c)).

The postsurgical healing was satisfactory, and complete removal was performed approximately two weeks after the incisional biopsy. Since OLP lesions remained unchanged and asymptomatic, no medications were prescribed, but follow-up was recommended. There was no evidence of recurrence after excision in a 7-year follow-up period, whereas the bilateral reticular OLP lesions on the buccal mucosa remained unchanged after the initial presentation.

## 3. Discussion

VX is a benign epithelial lesion that irrespective of intra- or extraoral development can simulate benign and malignant lesions causing diagnostic dilemmas [1–3]. The etiology remains obscure; the possibility of an association with lipid metabolism abnormalities was strongly speculated, but it has not been established [1–4, 13, 17]. The papillary morphology suggested the potential of HPV implication, but this was not confirmed by the results of several studies using immunohistochemistry or in situ hybridization methods [6, 24].

The total number of oral verruciform xanthomas with concomitant lichen planus reported in the literature including the present case amounts to twelve. However, in the studies of Yu et al. [4], Ide et al. [7], and de Andrade et al. [13], there were no sufficient data about the demographic, clinical, or histologic characteristics of the OLP and VX. The above three cases [4, 7, 13] were omitted from Table 1. The patients' age ranged between 42 and 73 years, and females were more frequently affected (6/9). Hume [17] described the first case of VX in a patient with clinical OLP features. In a patient with neurofibromatosis reported by Anbinder et al. [20], VX was located in the labial mucosa, while the diagnosis of OLP was based on clinical criteria alone. Including our case, the histologically confirmed cases of VX adjacent to OLP lesions are 4 [18, 19], while in two patients, VX did not develop in proximity to OLP lesions [19, 21]. The reticular type of OLP was present in every single case, while erosions (erosive OLP) [18], white plaques (hypertrophic OLP) [19], or erythema (atrophic OLP) [21] were also present in three cases. In our case, both the reticular and the hypertrophic OLP types were present.

The masticatory mucosa (gingival and alveolar mucosae) is referred as the most common intraoral site for VX. Traumatic or other unknown local predisposing factors may account for the frequent occurrence of this lesion in the gingival tissues [1–5, 7, 13]. On the other hand, the literature data on VX cases associated with OLP (Table 1) disclosed that nonkeratinized mucosa sites (tongue, buccal, or labial mucosa) are most frequently involved. Given the fact that these oral sites are affected frequently by OLP, this could suggest that the inflammatory infiltration in OLP may play a pathogenic role in the development of VX. OLP is characterized by basal cell degeneration, apoptotic keratinocytes in association with a chronic T-cell-mediated infiltrate, which maintains a condition of repeated keratinocyte damage/destruction and a consequent change in epithelial turnover [18]. The epithelial cell membranes releasing lipids are taken up by macrophages in the connective tissue becoming foamy in appearance, which eventually leads to VX development [1, 3, 7, 11]. A causal relationship between OLP and VX seems unlikely [19], since OLP is a relatively common oral mucosal disease, in contrast to VX. An accidental coexistence of VX and OLP cannot be excluded, but the VX may also occur concomitantly with autoimmune diseases associated with the chronic inflammatory process and epithelial cell damage/destruction, such as pemphigus vulgaris [8], lupus erythematosus [9], or graft-versus-host disease (GVHD) [11]. VX may represent an atypical T-cell-mediated local reaction to different aetiological agents related to degenerative epithelial changes [7]; such a viewpoint may be supported by the immunologic mechanisms predominated by T lymphocytes, which have been shown to take place during the VX development [2, 25].

VX considered as a reactive process may be involved in other pathologic conditions, such as dystrophic epidermolysis bullosa, in which skin or mucosa blistering may occur after minor trauma [10], or neoplastic lesions (carcinoma), in which the epithelial turnover may be affected [12, 13]. In CHILD syndrome, the formation of lipid-laden cells in VX may be related to this genetic disorder of cholesterol metabolism [14].

VX demonstrates a pathognomonic histological profile consisting of abundant, lipoid-rich macrophages with foam cytoplasm between the elongated uniform depth rete ridges. These cells are characterized by PAS-positive, diastase-resistant granules in their cytoplasm and display positive immunoreaction for the monocyte-macrophage markers CD68 and cathepsin B [2–7, 13, 23]. Three different histological patterns have been described based on the histological architecture and morphology of the lesion: verrucous or warty (A), papillary or cauliflower (B), and flat (C) or slightly raised [5]. The papillary pattern shows a finger-like exophytic epithelial proliferation covering thin cores of connective tissue, whereas in the flat pattern, the lesion demonstrates “endophytic” (below the surface) growth. The number of the foamy cells (xanthoma cells) may be related to the VX pattern. For example, in the flat pattern, numerous foamy cells accumulation can be observed in the lamina propria, possibly leading to the rete ridge elongation and thinning of the covered oral epithelium through compression [2]. In our case, the diagnosis of VX was based on the histopathological features characterized by epithelial hyperplasia in a verrucous pattern and the acanthotic thickened rete ridges combined with the foamy cell aggregations that exhibited strong immunoreactivity for the macrophage marker CD68 [1, 3, 5]. According to the histopathological pattern classification [5], the oral VXs with concomitant OLP reported in the literature (Table 1) exhibited more frequently the flat pattern(4/7), followed by verrucous (2/7) and papillary (1/7).

A correlation between the three histological patterns and the location of the lesions, the patients' age, or the biologic behavior of VX has not been referred in the literature [4, 5, 13]. Oral VX has a benign course with excellent prognosis after complete surgical excision. Recurrences are rare [3, 20].

## 4. Conclusions

The clinicians should be aware that clinically, verruciform xanthoma may mimic malignancy. Biopsy is required for definitive diagnosis to be established especially when this benign tumor occurs in conjunction with lesions or conditions that may exhibit the potential of malignant transformation, such as oral lichen planus, or may occur at high-risk sites for squamous cell carcinoma development, such as the tongue.

## Figures and Tables

**Table 1:** Reports of verruciform xanthoma occurring in patients with diagnosis or clinical evidence of oral lichen planus in the literature.

Study	Cases	Age/sex	VX location	Size (cm)	Histological pattern	OLP type	Association of VX with OLP lesions
Neville and Weathers [16]	1	67/F	Base of the tongue	1.5	–	NS	Patient with a history of OLP
Hume et al. [17]	1	55/F	Buccal mucosa at the occlusal level	1	–	NS	Clinical evidence of OLP
Miyamoto et al. [18]	1	68/F	Lateral aspect of the tongue	1.3	Verrucous	Reticular-erosive	VX occurring within OLP lesion
Polonowita et al. [19]	3	65/M, 73/F, 42/F	Gingiva, alveolar mucosa, lateral aspect of the tongue	NS (3)	Flat (3)	Reticular (2) and reticular-hypertrophic (1)	VX occurring within OLP lesion (2) and VX at an independent oral site (1)
Anbinder et al. [20]	1	70/M	Labial mucosa	NS	Flat	Reticular	Clinical evidence of lichen planus
Stoopler et al. [21]	1	68/M	Lateral aspect of the tongue	0.5	Papillary	Reticular-atrophic	VX occurring in the site adjacent to OLP lesion
Present case	1	56/F	Lateral aspect of the tongue	1	Verrucous	Reticular-hypertrophic	VX occurring within OLP lesion

NS: not specified; the classification of VX into 3 patterns according to the histological architecture appearance of the lesion was made in 1981 [5].

## References

[1] Neville B. W., Damm D. D., Allen C. M., Chi A. C., Neville B. W., Damm D. D., Allen C. M., Chi A. C. (2016). Epithelial pathology. *Oral and Maxillofacial Pathology*.

[2] Mostafa K. A., Takata T., Ogawa I., Ijuhin N., Nikai H. (1993). Verruciform xanthoma of the oral mucosa: a clinicopathological study with immunohistochemical findings relating to pathogenesis. *Virchows Archiv A Pathological Anatomy and Histopathology*.

[3] Philipsen H. P., Reichart P. A., Takata T., Ogawa I. (2003). Verruciform xanthoma-biological profile of 282 oral lesions based on a literature survey with nine new cases from Japan. *Oral Oncology*.

[4] Yu C. H., Tsai T. C., Wang J. T. (2007). Oral verruciform xanthoma: a clinicopathologic study of 15 cases. *Journal of the Formosan Medical Association*.

[5] Nowparast B., Howell F. V., Rick G. M. (1981). Verruciform xanthoma. A clinicopathologic review and report of fifty-four cases. *Oral Surgery, Oral Medicine, Oral Pathology*.

[6] Hu J. A., Li Y., Li S. (2005). Verruciform xanthoma of the oral cavity: clinicopathological study relating to pathogenesis. *APMIS*.

[7] Ide F., Obara K., Yamada H., Mishima K., Saito I., Kusama K. (2008). Cellular basis of verruciform xanthoma: immunohistochemical and ultrastructural characterization. *Oral Diseases*.

[8] Gehrig R. D., Baughman R. A., Collins J. F. (1983). Verruciform xanthoma in a young male patient with a past history of pemphigus vulgaris. *Oral Surgery, Oral Medicine, Oral Pathology*.

[9] Poulopoulos A. K., Epivatianos A., Zaraboukas T., Antoniades D. (2007). Verruciform xanthoma coexisting with oral discoid lupus erythematosus. *British Journal of Oral and Maxillofacial Surgery*.

[10] Murat-Susić S., Pastar Z., Dobrić I. (2007). Verruciform xanthoma in recessive dystrophic epidermolysis bullosa Hallopeau-Siemens. *International Journal of Dermatology*.

[11] Shahrabi Farahani S., Treister N. S., Khan Z., Woo S. B. (2011). Oral verruciform xanthoma associated with chronic graft-versus-host disease: a report of five cases and a review of the literature. *Head and Neck Pathology*.

[12] Drummond J. F., White D. K., Damm D. D., Cramer J. R. (1989). Verruciform xanthoma within carcinoma in situ. *Journal of Oral and Maxillofacial Surgery*.

[13] de Andrade B. A. B., Agostini M., Pires F. R. (2015). Oral verruciform xanthoma: a clinicopathologic and immunohistochemical study of 20 cases. *Journal of Cutaneous Pathology*.

[14] Bittar M., Happle R. (2004). CHILD syndrome avant la lettre. *Journal of the American Academy of Dermatology*.

[15] Fite C., Plantier F., Dupin N., Avril M. F., Moyal-Barracc M. (2011). Vulvar verruciform xanthoma: ten cases associated with lichen sclerosus, lichen planus, or other conditions. *Archives of Dermatolog*.

[16] Neville B. W., Weathers D. R. (1980). Verruciform xanthoma. *Oral Surgery, Oral Medicine, Oral Pathology*.

[17] Hume W. J., Smith C. J., Franklin C. D. (1980). Verruciform xanthoma. *British Journal of Oral Surgery*.

[18] Miyamoto Y., Nagayama M., Hayashi Y. (1996). Verruciform xanthoma occurring within oral lichen planus. *Journal of Oral Pathology and Medicine*.

[19] Polonowita A. D., Firth N. A., Rich A. M. (1999). Verruciform xanthoma and concomitant lichen planus of the oral mucosa. A report of three cases. *International Journal of Oral and Maxillofacial Surgery*.

[20] Anbinder A. L., Quirino M. R., Brandao A. A. (2011). Verruciform xanthoma and neurofibromatosis: a case report. *British Journal of Oral and Maxillofacial Surgery*.

[21] Stoopler E. T., Desai B. (2012). A tongue mass in a patient with oral lichen planus. *Journal-Canadian Dental Association*.

[22] Aghbari S. M. H., Abushouk A. I., Attia A. (2017). Malignant transformation of oral lichen planus and oral lichenoid lesions: a meta-analysis of 20095 patient data. *Oral Oncology*.

[23] Cheng Y. S., Gould A., Kurago Z., Fantasia J., Muller S. (2016). Diagnosis of oral lichen planus: a position paper of the American Academy of Oral and Maxillofacial Pathology. *Oral Surgery, Oral Medicine, Oral Pathology and Oral Radiology*.

[24] Iamaroon A., Vickers R. A. (1996). Characterization of verruciform xanthoma by in situ hybridization and immunohistochemistry. *Journal of Oral Pathology and Medicine*.

[25] Oliveira P. T., Jaeger R. G., Cabral L. A., Carvalho Y. R., Costa A. L., Jaeger M. M. (2001). Verruciform xanthoma of the oral mucosa. Report of four cases and a review of the literature. *Oral Oncology*.

